# Quantitative imaging of lipids in live mouse oocytes and early embryos using CARS microscopy

**DOI:** 10.1242/dev.129908

**Published:** 2016-06-15

**Authors:** Josephine Bradley, Iestyn Pope, Francesco Masia, Randa Sanusi, Wolfgang Langbein, Karl Swann, Paola Borri

**Affiliations:** 1 Cardiff School of Biosciences, The Sir Martin Evans Building, Museum Avenue, Cardiff CF10 3AX, UK; 2 Cardiff School of Physics and Astronomy, The Parade, Cardiff CF24 3AA, UK; 3 Cardiff University School of Medicine, Sir Geraint Evans Building, Heath Park, Cardiff CF14 4XN, UK

**Keywords:** Oocyte, Embryo, Egg, Microscopy, Lipid

## Abstract

Mammalian oocytes contain lipid droplets that are a store of fatty acids, whose metabolism plays a substantial role in pre-implantation development. Fluorescent staining has previously been used to image lipid droplets in mammalian oocytes and embryos, but this method is not quantitative and often incompatible with live cell imaging and subsequent development. Here we have applied chemically specific, label-free coherent anti-Stokes Raman scattering (CARS) microscopy to mouse oocytes and pre-implantation embryos. We show that CARS imaging can quantify the size, number and spatial distribution of lipid droplets in living mouse oocytes and embryos up to the blastocyst stage. Notably, it can be used in a way that does not compromise oocyte maturation or embryo development. We have also correlated CARS with two-photon fluorescence microscopy simultaneously acquired using fluorescent lipid probes on fixed samples, and found only a partial degree of correlation, depending on the lipid probe, clearly exemplifying the limitation of lipid labelling. In addition, we show that differences in the chemical composition of lipid droplets in living oocytes matured in media supplemented with different saturated and unsaturated fatty acids can be detected using CARS hyperspectral imaging. These results demonstrate that CARS microscopy provides a novel non-invasive method of quantifying lipid content, type and spatial distribution with sub-micron resolution in living mammalian oocytes and embryos.

## Abstract (precis)

**Summary:** CARS microscopy provides a novel and non-invasive method of quantifying lipid content, type and spatial distribution in living mouse oocytes and pre-implantation embryos.

## INTRODUCTION

The metabolism of mammalian oocytes and pre-implantation embryos is crucially dependent upon >100,000 mitochondria (Acton et al., 2004). Pyruvate in the media around the oocyte provides a major source for oxidative metabolism in the mitochondria. However, fatty acid (FA) metabolism is another substantial energy source for mitochondria and is particularly notable in oocytes of domesticated animals, such as pigs, that contain very large lipid droplets (LDs) (McEvoy et al*.,* 2000). FA metabolism appears to be essential for preimplantation development in all mammalian embryos, including those with less lipid content (Downs et al., 2009; Dunning et al., 2010). The amount or type of FA, whether saturated or unsaturated, to which embryos are exposed affects their development capacity (Aardema et al., 2011). The FA composition of human follicular fluid has been shown to predict the outcome of pregnancies in human *in vitro* fertilisation (IVF) (Shaaker et al., 2012). This suggests that measuring the amount and type of FA in mammalian oocytes or embryos could be a key tool in both research and clinical studies of mammalian development. Notably, the lipid content of oocytes varies considerably between species. In the two most studied and noteworthy species, namely mice and humans, oocytes and embryo lipid content is relatively low, and LD sizes require sub-micron-resolution imaging techniques to be resolved (Watanabe et al., 2010).

The lipid content of mammalian oocytes and embryos has traditionally been assayed by destructive chemical analysis (Ferreira et al., 2010; McEvoy et al., 2000; Loewenstein and Cohen, 1964). Alternatively, LDs have been imaged in mammalian oocytes by staining with dyes such as Nile Red or BODIPY 493/503 (Genicot et al., 2005; Yang et al., 2010). These fluorescent stains provide only a semi-quantitative assay of lipid content because of their limited specificity, often uncontrolled variability in fluorophore densities, and the limitations arising from photobleaching. Furthermore, staining with such dyes is incompatible with oocyte maturation or embryo development and is usually carried out on fixed samples. Label-free imaging techniques have attracted increasing attention recently, in order to overcome these limitations. To that end, vibrational Raman confocal micro-spectroscopy (based on the interaction of light with vibrations of endogenous chemical bonds) has been successful in imaging LDs label-free in mouse eggs. However, Raman scattering is a very weak process, and the long image acquisition times needed to generate sufficient contrast have again effectively limited its use to fixed material (Wood et al., 2008). Furthermore, mammalian oocytes and embryos are particularly sensitive to light, hence light exposure has to be minimised in order to maintain viability (Takenaka et al., 2007). Recently, third-harmonic generation (THG) microscopy has been used to image LDs label-free in mouse embryos, in a way that is compatible with subsequent development (Watanabe et al., 2010). However, THG is sensitive to interfaces rather than chemical content. It allows morphological imaging of small structures, but does not provide quantitative information on the amount and type of lipids, and did not appear to resolve individual LDs in Watanabe et al*.* (2010). Consequently, there are no methods reported to date for quantitatively assessing lipid content in mammalian oocytes and early embryos in a non-destructive manner. This precludes time-course studies of lipid metabolism in the same embryos that are assessed for development. It also prevents any potential use of LDs as a predictor of oocyte quality or embryo developmental potential.

CARS microscopy has emerged in the last decade as a powerful multi-photon microscopy technique that overcomes some limitations of spontaneous Raman scattering and enables label-free chemical and quantitative analysis of lipids at high imaging speeds in living cells (for a recent review see Zumbusch et al., 2013). Briefly, CARS arises as a result of a third-order nonlinear process (four-wave mixing) in which two near-infrared pulsed laser fields of frequencies ν_P_ (pump) and ν_S_ (Stokes) coherently excite a molecular vibration resonant at the frequency difference ν_vib_=ν_P_−ν_S_. The CARS field at the frequency 2ν_P_−ν_S_=ν_P_+ν_vib_ is generated from the modulation of the pump field by this coherent vibration. The CARS frequency is higher than the exciting field frequencies and thus free from (one-photon) auto-fluorescence background. Importantly, CARS benefits from the constructive interference of the Raman signal generated by all vibrational modes of a given type within the focal volume, resulting in intensities several orders of magnitude higher than spontaneous Raman. This is particularly beneficial for imaging lipids as they contain a large number of identical CH bonds in the FA chain. Moreover, the nonlinear CARS process only takes place in the focal volume where high photon densities are reached, allowing for an intrinsic three-dimensional (3D) spatial resolution without the need of a confocal detection pin-hole. As well as acquiring images at one particular vibrational frequency, it is also possible to perform hyperspectral CARS imaging, which provides a CARS spectrum at each spatial position. This enables the quantitative analysis of the amount and chemical composition of FAs in cells (Rinia et al., 2008; Di Napoli et al., 2014b). CARS has previously been used to image LDs in living cells in culture and invertebrate embryos (Hellerer et al., 2007; Zumbusch et al., 2013), but such cells or embryos generally tolerate much higher levels of incident light intensities than mammalian oocytes (Takenaka et al., 2007).

In this paper, we demonstrate quantitative CARS imaging applied to living mouse oocytes and early embryos. We show that we can resolve individual LDs and quantify their 3D spatial distribution label-free. Importantly, we find the degree of aggregation of LDs significantly changes during oocyte maturation and during embryo pre-implantation development, suggesting that it could provide an indicator of developmental potential. Notably, we show that CARS imaging can be compatible with oocyte maturation and development to blastocyst. By performing simultaneous CARS and two-photon fluorescence (TPF) microscopy on the same oocytes labelled with conventional fluorescent lipid probes, we find good correlation between LDs from CARS imaging with those from TPF images using BODIPY 493/503, but poor correlation when using LipidTOX. In addition, we show that hyperspectral CARS microscopy can be used to assess differences in the chemical composition of LDs of living mouse oocytes matured in media supplemented with different saturated and unsaturated FAs. This work demonstrates a non-invasive methodology based on label-free quantitative imaging of LD spatial distribution and lipid content, which has the potential to provide a new tool to assess oocyte quality and pre-implantation embryo viability in mammals.

## RESULTS

### CARS imaging of lipid droplets in mouse eggs and embryos

**Fig. 1.**
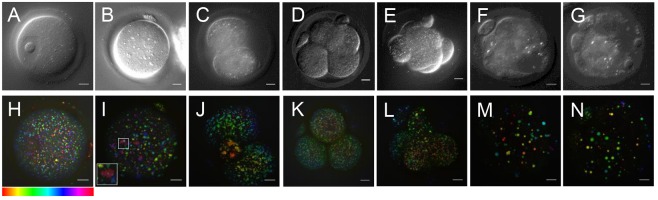
**CARS and DIC images in living oocytes, eggs and early embryonic stages.** (A-G) DIC images (single *z*-plane, except E, which is a maximum intensity projection) representative of populations of mouse eggs and embryos, from (A) immature GV stage (*n*=∼90), (B) MII eggs (*n*=∼70), (C) two-cell (*n*=∼65), (D) four-cell (*n*=∼60), (E) eight-cell (*n*=∼10), (F) morula (*n*=∼35) and (G) blastocyst stage (*n*=∼20) embryos using a 1.27 NA water objective and a 1.4 NA oil condenser. (H-N) Depth colour-coded images of CARS *z*-stacks at wavenumber 2850 cm^−1^ through the same eggs and embryos, showing LDs throughout these developmental stages. Inset in (I) shows a typical LD cluster seen at this stage. 0.1×0.1 µm *xy* pixel size; 0.5 µm *z*-step; 0.01 ms pixel dwell time; ∼14 mW (∼9 mW) pump (Stokes) power at the sample. Scale bars: 10 µm. Colour bar shows depth colour-coding from –25 µm-25 µm of 101 *z*-stacks (0 µm being the approximately equatorial plane of the egg or embryo), the brightness of each colour is the maximum intensity at each corresponding *z*-plane. Data from ≥2 trials, using 1-3 mice each.

The CH_2_ symmetric stretch vibration at ∼2850 cm^−1^ is abundant in the acyl chain of FAs, and is an ideal target for single-frequency CARS microscopy of LDs. Living mouse oocytes, eggs and early embryos were imaged label-free using differential interference contrast (DIC) and CARS microscopy. Fig. 1A-G shows DIC images in a single, approximately equatorial *z*-plane of all developmental stages, from germinal vesicle (GV) stage through to a blastocyst embryo, in a set of representative specimens, each showing stage-specific characteristics. Accompanying each DIC image is a corresponding 3D CARS image of the same sample, acquired immediately after DIC imaging. These are shown as depth colour-coded CARS *z*-stack projections, measured at the 2850 cm^−1^ CH_2_ symmetric stretch vibrational resonance, and enabled us to determine the LD spatial distribution throughout the measured volume at each stage. LDs in a GV oocyte have a homogenous spatial distribution throughout the cell cytoplasm, but not within the GV itself (Fig. 1H). The distribution of LDs in metaphase II (MII) eggs is noticeably different, with many clusters of LDs seen throughout the cytoplasm, and fewer dispersed singular droplets (Fig. 1I). CARS images of early embryo stages (two-cell, four-cell embryos) show that LDs fill most of the cytoplasm as the cells divide, with little or no difference in distribution between blastomeres (Fig. 1J,K). LDs appear of uniform size, ∼0.5 µm diameter (see Fig. S1); however, by the eight-cell stage, larger droplets are seen, and by the later early-embryo stages (morula and blastocyst) fewer, but much larger (>1 µm) LDs are present, varying in size, suggesting fusion of existing droplets as development continues through these stages (Fig. 1L-N).

**Fig. 2.**
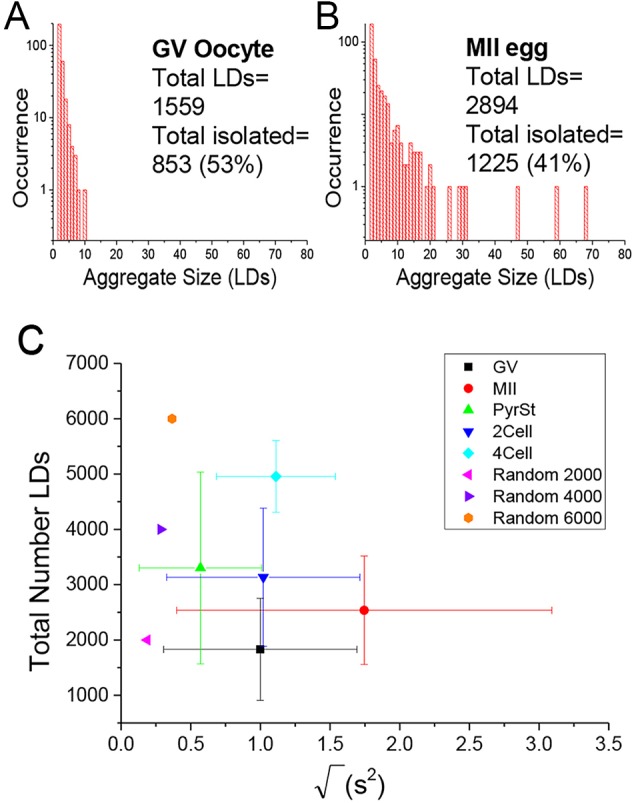
**Lipid droplet quantification and clustering analysis.** (A,B) Histograms of LD aggregate sizes (number of LDs in each cluster, and their occurrence) in a typical (A) GV oocyte, and (B) MII egg. Total number of LDs and total number of un-clustered LDs are also indicated. (C) Scatter plot of the square root of the mean square aggregate size (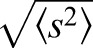) against the total number of LDs, in ensembles of GV oocytes (*n*=33), MII eggs (*n*=30), MII eggs starved of pyruvate (PyrSt; *n*=8), two-cell (2Cell; *n*=10) and four-cell (4Cell; *n*=8) embryos (including those represented in Fig. 1). The distribution of each variable in the corresponding ensemble is shown as a mean (symbol) and standard deviation (bar). The case of a random LD distribution simulated for a range of total number of LDs is also shown for comparison.

Using in-house developed software, the coordinates of each droplet were determined by fitting the three-dimensional intensity distributions of each droplet within the CARS images. This method enabled us to count the total number of LDs within the measured sample volume, and provided the size of each droplet (width in *x*, *y* and *z*) as fit parameters. The coordinates were then used to determine whether each individual LD was part of an aggregate (two or more LDs), or was an isolated droplet. A LD was considered part of an aggregate if separated from its nearest neighbour by a distance less than 1.5 times the optical resolution. By then calculating the occurrence of each aggregate size (number of LDs per aggregate), we found that out of all eggs imaged, MII eggs have the most clusters made up of 10 or more droplets (see examples in Fig. 2A,B). To provide statistical significance, we examined a set of *n>8* oocytes and embryos at each stage of development and calculated histograms as shown in Fig. 2A and B for each oocyte and embryo. To compare these groups, we calculated the mean aggregate size 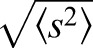 (see supplementary Materials and Methods) from the histogram of each oocyte and embryo (including those shown in Fig. S1), and plotted these values per egg or embryo population in a two-dimensional plot against the total number of LDs, where the distribution of these two variables for each group is represented as a mean (symbol) and standard deviation (bar) (Fig. 2C). There is substantial variability within each group, but the MII group is distinguished through large values of 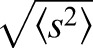. Notably, the four-cell group is also distinguished through a larger total number of LDs. We also calculated that there was a statistically significant difference in the percentage of aggregated droplets between the GV and MII stages of oocyte development using a *t*-test (result of *P*=0.04, data not shown). For comparison, we simulated the case of random LD distribution, with a total number of droplets within the ranges observed experimentally. This is plotted in Fig. 2C (magenta, violet and orange triangles) and shows that the average 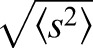 is below that observed experimentally in each group, indicating that even when less aggregating in the GV stage, LDs still show more aggregation than expected from a random distribution. Notably, starving MII eggs of pyruvate resulted in a dispersion of LD aggregates [also shown in Fig. 2C, green triangles (PyrSt) and Fig. S2], resembling the random distribution case, and suggesting a link between the metabolic use of FAs and LD spatial distribution.

**Fig. 3.**
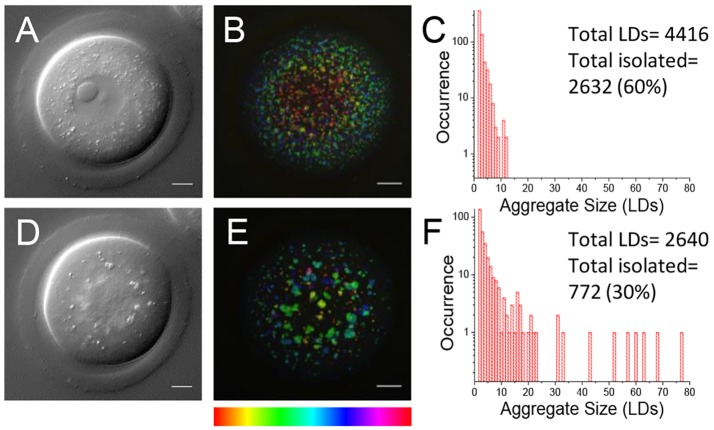
**Cell viability after live imaging with CARS.** (A,D) Single *z*-plane DIC using a 1.27 NA water objective and a 1.4 NA oil condenser and (B,E) depth colour-coded images of CARS stacks at wavenumber 2850 cm^−1^, of an egg before and after *in vitro* maturation, showing that development can still occur after live imaging with CARS (*n*=40). (C,F) Histograms of the number of LDs making up clusters in these cells, demonstrating the change in LD distribution over time. 0.1×0.1 µm *xy* pixel size; 0.5 µm *z*-step; 0.01 ms pixel dwell time; ∼13 mW (∼9 mW) pump (Stokes) power at the sample. Scale bars: 10 µm. Colour bar shows depth colour-coding from –25 µm-25 µm (0 µm being the equatorial plane). Data from >5 trials, using 1-3 mice each.

We investigated in detail the excitation conditions in our CARS experiment that allowed for live-cell imaging. We found that immature GV oocytes matured to MII stage *in vitro* after CARS imaging of the whole cell under the excitation conditions as in Fig. 1 (40/47 oocytes matured). Fig. 3 shows a CARS image of a fully scanned GV oocyte prior to *in vitro* maturation (IVM), and the same cell after 18 h in culture, once it had reached a mature stage. Notably, before maturation (Fig. 3A-C) the GV oocyte had a widely dispersed LD spatial distribution, and after IVM (Fig. 3D-F) the same cell is seen as an MII egg with characteristically aggregated LDs confirmed by quantitative analysis (Fig. 3C,F), supporting the observations from Fig. 1, in this case on the same egg followed over time. Control eggs that were not imaged prior to IVM, along with MII eggs matured *in vivo*, are included in Fig. S3.

**Fig. 4.**
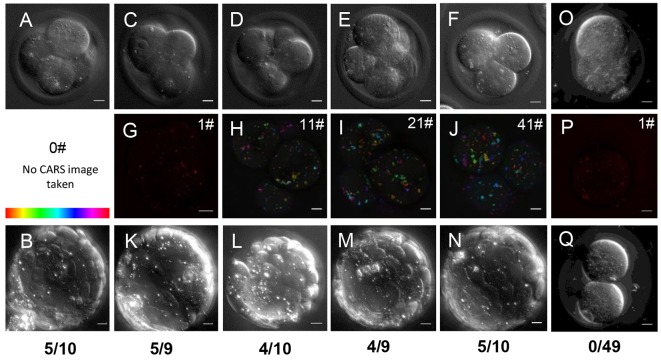
**Embryo viability after live imaging with CARS.** (A) Single *z*-plane and (B) maximum intensity projection DIC images taken with a 1.27 NA water objective and a 1.4 NA oil condenser of an embryo at four-cell stage and blastocyst stage, without CARS imaging (*n*=10). (C-F) Single *z*-plane DIC and (G-J) depth colour-coded CARS stacks of four-cell embryos before their development to blastocyst stage after different CARS imaging at 2850 cm^−1^. Number of CARS images taken is indicated in the top right corner; (G) 1 CARS *xy* image (*n*=9), (H) 11 CARS *xy* images with 5 µm z-steps (*n*=10), (I) 21 CARS *xy* images with 0.5 µm *z*-steps (*n*=9), (J) 41 CARS *xy* images taken with 0.5 µm *z*-steps (*n*=10). (K-N) Maximum intensity projection DIC images of blastocyst stages of the same embryos seen at the four-cell stage in panels C-J; blastocyst developmental rate is indicated beneath panels. (O,P) DIC and CARS (1 *xy*) images of a two-cell embryo, and (Q) DIC image of the same embryo 5 days later, demonstrating arrest (*n*=49). 0.1×0.1 µm pixel size; 0.01 ms pixel dwell time; ∼14 mW (∼9 mW) pump (Stokes) power at the sample. Scale bars: 10 µm. Colour bar shows depth colour-coding from –25 µm-25 µm (0 µm being the equatorial plane). Data from >5 trials, using 1-3 mice each.

Conversely, CARS imaging with the same parameters at the two-cell stage seemed to hinder development to later embryonic stages, even when single *z*-planes were imaged. One hundred percent arrest at two-cell stage was observed (*n*=49; Fig. 4O,P) in comparison to 15/24 developing to blastocyst in the un-imaged control group. Imaging from the four-cell stage onwards – a much less vulnerable stage of development than two-cell in mouse embryos (Qiu et al*.,* 2003) – allowed development to blastocyst stage at the normal rate of 50%, as seen in humans and the particular strain of mouse used, specifically chosen for this developmental similarity (Kamjoo et al*.,* 2002). A number of experimental step-size settings were investigated; all images were taken with imaging parameters previously described (see Materials and Methods). Embryos that were imaged with CARS every 0.5 μm in *z* over both a 20 μm and a 10 μm *z*-range all continued to develop further, 5/10 and 4/9 reaching a recognisable blastocyst stage, respectively (Fig. 4E,I,M and F,J,N). Those imaged more sparsely, every 5 μm in *z* over a 50 μm *z*-range all developed and 4/10 were classed as blastocysts (Fig. 4D,H,L). Taking a singular CARS image at the centre of the four-cell embryo, or imaging solely with DIC gave rise to a blastocyst yield of 5/9 and 5/10, respectively (Fig. 4A,B and C,G,K), giving the same rate of development as a control group that was not imaged (5/10). To examine whether pre-implantation development was possible after imaging at the one-cell stage we also took CARS images of zygotes fertilised by ICSI (intra-cytoplasmic sperm injection) (Yoshida and Perry, 2007). ICSI is more successful in oocytes where cumulus cells have been removed for imaging, and is widely used in reproductive biology. CARS images from fertilised zygotes (see Fig. S4) lead to 13/34 blastocysts, which compared with 5/30 blastocysts in control embryos. The difference between these developmental rates was not significant but higher rates of development in the CARS-imaged group suggest that such imaging does not impair pre-implantation development.

### Comparison between CARS imaging and fluorescent staining of lipid droplets

**Fig. 5.**
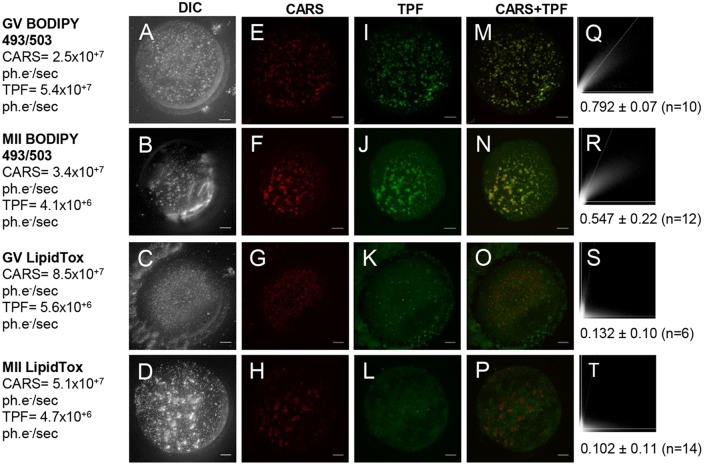
**CARS images compared with conventional fluorescent lipid dyes on fixed eggs.** (A-D) DIC (using a 1.27 NA water objective and a 1.4 NA oil condenser), (E-H) false-coloured CARS images at wavenumber 2850 cm^−1^ and (I-L) TPF *xy* images, accompanied by (M-P) false-coloured overlays, of GV and MII eggs stained with BODIPY (*n*=23 and 30, respectively) or LipidTox green neutral lipid stain (*n*=7 and 20, respectively). All images are maximum intensity projections of stacks with 0.5 µm *z*-steps. 0.1×0.1 µm pixel size; 0.01 ms pixel dwell time; ∼12 mW (∼9 mW) pump (Stokes) power at the sample. CARS and TPF signal intensities are given in photoelectrons per second (ph.e^−^/s). Scale bars: 10 µm. (Q-T) Scatterplots of pixel-coordinate correlation between CARS (*x*-axis) and TPF images (*y*-axis), and the mean Pearson's correlation coefficients of all investigated eggs, to show the degree of reliability of these dyes. A colocalisation is apparent with a Pearson's coefficient of >0.5, but is only considered significant if >0.95 as in accordance with normal 95% confidence limits. Data from ≥4 trials, using 1-3 mice each.

BODIPY 493/503 and LipidTOX are green-emitting neutral lipid stains commonly used to investigate LD biology. However, by comparison with CARS microscopy it has been shown by several groups that these or similar lipid dyes (e.g. Nile Red) do not specifically stain LDs, for example in *Caenorhabditis elegans* (Hellerer et al., 2007; Klapper et al., 2011; Le et al., 2010). In order to examine how BODIPY 493/503 and LipidTOX stain LDs in mouse eggs, we used two-photon fluorescence (TPF) alongside simultaneous CARS measurements in fixed oocytes with fluorescently labelled LDs. Both dyes, when incubated as per manufacturer's instructions, stained more structures than solely LDs within the egg cytoplasm, demonstrated clearly when overlaying TPF images with false-coloured CARS images taken simultaneously (Fig. 5). In some cases, intracellular vesicles that are not LDs were also stained, and in other cases, LDs revealed by CARS were not stained. Simultaneous detection (via a third photomultiplier) in a spectral range blue-shifted from the green emission band of the lipid dyes, showed autofluorescence from multiple cellular organelles (albeit ∼15 times lower than the fluorescence dye emission), showing that these fluorescence methods are not free from background, and are not selective enough to be reliably used to quantify lipid content of mouse oocytes. Correlation studies of the CARS versus TPF images gave a more quantitative analysis of the specificity of these stains. We used Pearson's correlation coefficient analysis where values of >0.5 indicate recognisable co-localisation. An average Pearson's correlation coefficient of 0.132 (GV) and 0.102 (MII) with LipidTOX staining indicates no real co-localisation between CARS and TPF images (Fig. 5S,T). Using BODIPY 493/503 instead showed moderate co-localisation with an average correlation coefficients of 0.792 (GV) and 0.547 (MII) (Fig. 5Q,R).

### Analysis of chemical composition with hyperspectral imaging

Compared with single-frequency CARS, hyperspectral CARS microscopy provides more specific information on the chemical composition of LDs, including their degree of saturation, by acquiring a spectrum of CARS frequencies for each spatial point (Masia et al., 2013, Di Napoli et al., 2014a). In order to be compatible with living eggs and embryos, we acquired single *xy* images at 1 µs pixel dwell time (ten times less than for the single frequency images, but with equal power) across the CH-stretch frequencies (2600-3800 cm^−1^, 5 cm^−1^ step) to obtain vibrational spectra of LDs in different spatial positions of eggs and embryos at different developmental stages.

**Fig. 6.**
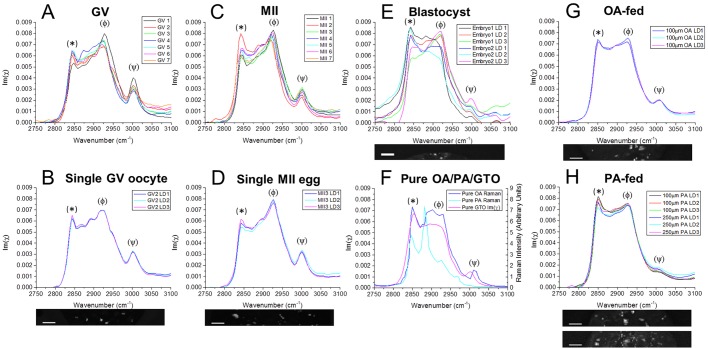
**Hyperspectral imaging of chemical content of oocytes, eggs and embryos.** (A-E) Vibrational Raman-like spectra Im(χ) obtained from CARS hyperspectral images. (A) Example LDs in all GV oocytes (*n*=7) and (B) three LDs in the same GV oocyte, shown in the accompanying image. (C) Example LDs in all MII eggs (*n*=7) and (D) three LDs in the same MII egg, shown in the accompanying image. (E) Three LDs in two different blastocyst stage embryos, ‘Embryo 2’ shown in the accompanying image. (F-H) Raman spectra of (F) pure oleic acid (OA) and palmitic acid (PA) in the solid (ordered) phase (digitised from spectra given by Sigma), against the retrieved PCKK spectrum of glycerol trioleate (GTO; oleic acid in its triglyceride form), (G) three LDs in an MII egg *in vitro* matured in 100 µM oleic acid (*n*=16), and (H) three LDs in MII eggs *in vitro* matured in 100 µM (*n*=31) or 250 µM palmitic acid (*n*=33), LDs shown in the accompanying images. Spectra are normalised to the total area. Peaks: (*) ∼2850 cm^−1^ correspond to the symmetric CH_2_ stretch; ∼2880 cm^−1^ to the asymmetric CH_2_ stretch, especially enhanced in ordered/solid-phase; (φ) ∼2930 cm^−1^ to CH_3_ and asymmetric CH_2_ stretch vibrations, enhanced in disordered/liquid-phase acyl chains; (ψ) ∼3010 cm^−1^ correspond to the =CH stretch. Note, the intensity ratio between bands at 2880 cm^−1^ and 2850 cm^−1^ can be used as a measure of acyl chain order; the ratio between peaks at 2930 cm^−1^ and 2850 cm^−1^ can be used to ascertain chain disorder. Accompanying images show the 10 µm×80-100 µm area over which hyperspectral scans were obtained. Scale bars: 10 µm. Data from ≥2 trials, using 1-3 mice each.

Hyperspectral CARS images of LDs in GV oocytes and MII eggs were analysed with software developed in-house to retrieve Raman-like spectra by determining the imaginary part of the complex CARS susceptibility at each spatial point (Masia et al., 2013, 2015). The shape of the retrieved vibrational spectra at the LD positions in both developmental stages is characteristic of polyunsaturated FAs (Fig. 6A-D,F), with a prominent band at ∼2930 cm^−1^ from the CH_3_ stretch and asymmetric CH_2_ stretch vibrations enhanced by the broadening and shift of the CH deformations in the liquid disordered phase, a less-prominent peak at ∼2850 cm^−1^ corresponding to the symmetric CH_2_ stretch, and a third peak at ∼3010 cm^−1^ corresponding to the =CH stretch (Di Napoli et al., 2014a). Spectra were consistently of similar shape both across multiple LDs within the same cell (Fig. 6B,D) and across different cells (Fig. 6A,C).

Hyperspectral CARS imaging of blastocyst-stage embryos and analysis of their large LDs showed that within the same embryo, LDs had differing compositions of saturated and unsaturated FAs (Fig. 6E,F). Retrieved Raman-like spectra showed LD profiles similar to the GV and MII case, as well as droplets with a less prominent 2930 cm^−1^ band relative to the 2850 cm^−1^ peak characteristic of reduced FA unsaturation and increased chain order (Di Napoli et al., 2014a,b).

Furthermore, it was found that after incubation in FAs, the chemical composition of LDs reflects that of the FA supplements. Hyperspectral imaging was performed on MII eggs that had been *in vitro* matured overnight in differing concentrations of mono-unsaturated oleic acid (18:1) and saturated palmitic acid (16:0). After incubation with 100 µM oleic acid, the retrieved vibrational spectrum changed towards the Raman spectrum of mono-unsaturated oleic acid, or OA in its triglyceride form – glycerol trioleate (GTO) (Fig. 6F,G) – from the spectrum of polyunsaturated lipids originally observed in MII in Fig. 6C and D. After incubation with 100 or 250 µM palmitic acid, the spectrum tends to have an even more saturated FA-like line shape, with a more pronounced peak at 2850 cm^−1^ and reduced =CH resonance at ∼3010 cm^−1^ (Fig. 6F,H). These peak shifts are displayed quantitatively in Table S1. Note that pure palmitic acid is solid until 60°C, with the peak at 2880 cm^−1^ in the Raman spectrum being a signature of such solid phase, not expected in LDs consisting of mixed FAs. These data show that hyperspectral imaging using CARS microscopy can be used to distinguish differences in the chemical composition of LDs, specifically saturated versus unsaturated lipids, within living oocytes and embryos.

## DISCUSSION

In this study we have shown that CARS micro-spectroscopy is a useful tool to visualise LDs in mouse eggs and embryos at high spatial resolution in a label-free, non-invasive, and chemically specific manner. We were able to resolve individual LDs and provide quantitative information on the total number, size and distribution of LDs in 3D, as well as follow changes in these quantities throughout different stages of meiotic and mitotic development, on statistically relevant numbers of eggs and embryos.

Mammalian oocytes, eggs and cleavage stage embryos rely almost exclusively upon the metabolism of pyruvate and FAs by their mitochondria for generating energy in the form of ATP (Acton et al., 2004; Dumollard et al., 2008). It was notable that LDs containing FAs were more aggregated in mature MII eggs as opposed to immature GV stage oocytes, as quantified by aggregate analysis (see Fig. 2C). The dispersed spatial distribution (albeit not random) of the LDs at GV stage suggests an availability of FAs for metabolism at this stage. This is supported by the observation that starving MII eggs of pyruvate, hence forcing FA metabolism as the only remaining energy source, restored a highly dispersed distribution of LDs (even more dispersed than GV) with very few clusters compared with control MII eggs. However, it is not clear exactly how the difference between GV and MII stages is related to metabolic changes, because oxidative phosphorylation predominates at both stages (Dumollard et al., 2008). Indeed, it is possible that the large variability in the LD distribution observed within the GV and MII groups is a consequence of a balance between pyruvate in the media and FA metabolism. This can be affected by the quality and developmental potential of the oocytes, and requires further dedicated study. There appears to be a net increase in LD number as development continues (see Fig. 2C). This might result from the culture of embryos, and an increase in lipid synthesis resulting from the presence of BSA in the culture medium. Although there are studies of lipid content during development in domestic animals, as far as we are aware there are no studies quantifying lipid content in developing mouse embryos, so this effect would require further studies. Interestingly, we saw the appearance of larger LDs at the morula and blastocyst stage. Notably, around these stages of pre-implantation development there is a switch to the greater use of glycolysis to provide ATP and pyruvate. The larger LDs might therefore be indicative of a phase of FA storage and reduced β-oxidation.

It is noteworthy that in mammalian embryology, where there are limited samples that are of considerable variability, there is a particular need for simple, non-invasive techniques that can provide quantitative information on embryo viability so that biochemical parameters can be correlated with subsequent development (Ebner et al., 2003). We have shown that live CARS imaging of oocytes and embryos can be performed in a way that it does not harm their ability to develop to later embryonic stages at the usual rate (see Figs 3, 4 and Fig. S4). Although imaging embryos at the two-cell stage appears to halt their development, this is recognised as a vulnerable stage of mouse embryo development (Qiu et al., 2003). Instead, imaging a smaller portion of the embryo at the one-cell stage after fertilisation, or at the four-cell stage, was compatible with continued embryonic development. Further studies into the viability of these blastocysts would be necessary, such as monitoring implantation and birth of live offspring. However, for future analysis of LD distribution and content as a measure of embryonic developmental potential, only one CARS image or a fast scan through the cells might be sufficient to provide enough detail to make an informed assessment. It is possible that future studies of LD distribution and developmental potential could provide a potential diagnostic tool for selecting embryos in clinical IVF or animal reproductive technologies. CARS imaging using excitation at longer wavelengths than used in the present study (>900 nm, suppressing the three-photon absorption of DNA) is possible, and should allow for even lower phototoxicity and higher penetration depth. This can be combined with THG imaging, which can be used for non-chemically specific LD imaging, and stimulated Raman scattering (SRS) imaging, which reduces the background and allows better quantitative imaging when probing only a single vibrational frequency.

A number of lipid dyes have previously been used to stain LDs in mammalian oocytes, including Nile Red and BODIPY 493/503 (Yang et al., 2010; Del Collado et al., 2015; Genicot et al., 2005). Nile Red is commonly used on porcine and bovine oocytes that have large LDs (Sturmey et al., 2006; Leroy et al., 2005). Staining with such fluorescent dyes is incompatible with oocyte maturation or embryo development and it is usually carried out on fixed samples. Besides this obvious drawback, lipid staining also raises questions on specificity and reliability. By correlating chemically specific CARS imaging of LDs with TPF imaging of green-emitting lipid dyes in fixed MII eggs, we found that BODIPY 493/503 stains a number of vesicles that appear to overlap with LDs identified by CARS imaging; however, there was only a moderate correlation. We also correlated CARS imaging with TPF from another LD probe widely used in many somatic cells, LipidTOX, and found very weak correlation (see Fig. 5). We could not measure simultaneous CARS and TPF with Nile Red, as the excitation and emission spectrum of this red-emitting dye is not suited for our multimodal microscope, but we performed separate confocal fluorescence imaging (see Fig. S5). Nile Red seemed effective at staining vesicles; however, previous studies correlating Nile Red staining with CARS in *C. elegans* demonstrate its lack of specificity (Hellerer et al., 2007; Klapper et al., 2011). Overall, these data exemplify the limitations of LD staining specificity, and the advantage of label-free CARS imaging as a reliable and quantitative method for measuring LDs in live mammalian oocytes, eggs and early embryos.

It has been previously shown that exposure of oocytes to excess FAs affects the subsequent embryo development after fertilisation (Ferreira et al., 2010; McEvoy et al., 2000; Loewenstein and Cohen, 1964), and that positive or negative effects of lipids depend on the particular FAs present. Palmitic acid has been shown to have detrimental effects to the developing egg or embryo, whereas oleic acid has been found to have positive effects and even reverse those of palmitic acid, by promoting the formation of LDs. The type of FA found in follicular fluid has also been shown to be predictive of IVF success rates (Aardema et al., 2011; Shaaker et al., 2012). These data suggest that the type of lipid an oocyte is exposed to, or composed of, will influence viability. By performing CARS hyperspectral imaging of living mouse eggs and early embryos, we have been able to provide information on the chemical content of LDs, specifically with regards to the degree of saturation of FAs within the egg or embryo. We observe that LDs in the early oocyte and egg stages are composed of a high proportion of polyunsaturated FAs, and are quite homogeneous in composition between different LDs in the same cell. Conversely, the large LDs seen at blastocyst stage appear to contain less unsaturated FAs and be more heterogeneous. This has the potential to be an interesting diagnostic tool, as previous research shows that human embryos with higher concentrations of unsaturated FAs were more likely to develop beyond the four-cell stage (Haggarty et al., 2006). We also show that the actual LD FA composition can change when oocytes are exposed to saturated or unsaturated FAs in the same concentration range where effects upon embryo viability have been previously demonstrated (Aardema et al., 2011). When fed with a particular FA, the chemical composition of LDs resembles that of the FA supplements. Vibrational spectra are not identical to those of pure FAs because of the mixture of FAs still composing each droplet. Methods we describe here could be used in future studies of embryo quality; for example, it would be of interest to assess how the FA content changes with exposure to different environmental factors such as a high fat diet, which is known to have detrimental effect upon developmental potential (Wu et al., 2010).

## MATERIALS AND METHODS

### Gamete collection and manipulation

4-6-week-old female MF1 mice were intraperitoneally injected with 5 IU PMSG to induce ovarian follicle growth. Approximately 24 h later, immature GV oocytes were collected from ovarian follicles or 10 IU hCG was injected to cause superovulation, and oviductal mature (MII) eggs were collected 15 h later. All animals were handled according to UK Home Office regulations, and procedures carried out under a UK Home Office Project Licence. Cumulus cells were removed by gentle pipetting (GV) or brief exposure to hyaluronidase (MII). Oocytes and eggs were kept at 37°C in drops of M2 medium (embryo-tested, Sigma). To maintain GV arrest, 100 μM IBMX (Sigma) was included. IVM was initiated by washing and culturing oocytes in standard M2 medium at 37°C or Minimum Essential Medium (MEM; Sigma) containing 3 mg/ml bovine serum albumin (BSA; Sigma). Drops of media were covered with mineral oil (embryo-tested, Sigma) to prevent evaporation.

Pyruvate starvation was carried out by incubating MII eggs in HEPES-buffered KSOM medium containing 3 mg/ml BSA (both Sigma), lacking pyruvate and lactate, for ∼6 h before imaging.

*In vivo* fertilisation was carried out by mating female MF1 mice with 10-24-week-old male F1-hybrid mice after the hCG injection, and oviductal zygotes were collected 15 h later. Embryos were cultured in KSOM (Millipore) at 37°C, 5% CO_2_ under oil for up to 5 days, when blastocyst stage is reached.

Where stated, egg and embryos were fixed in 1% paraformaldehyde (PFA) in phosphate buffered solution (PBS) with 1% polyvinyl alcohol (PVA) to prevent adhesion, for 10 min before holding in PBS. Fixed cells were stained for LDs using 1 µg/ml BODIPY 493/503 or HCS LipidTOX green neutral lipid stain at a 1:5000 stock dilution (both Life Technologies).

Experiments were carried out on sample sizes of five or more oocytes or embryos for adequate representation, and repeated where possible. Eggs and embryos were randomly assigned to experimental and control groups, and only living eggs and embryos were included in data acquisition and analysis.

### CARS, DIC and TPF microscopy

Standard microscope slides were prepared with SecureSeal imaging spacers (Sigma) of ∼150 μm thickness. Petroleum jelly was thinly spread around the well of the spacer for an airtight seal. Eggs were pipetted into a small drop of ∼10-20 μl of M2 media, embryos into PBS with PVA, and a coverslip used to create a sealed chamber. For imaging requiring exchange or addition of media, living cells or embryos were pipetted into a drop of M2 media covered in oil, and placed in an in-house-built imaging dish with a 25 mm round glass coverslip bottom and a removable glass lid.

The CARS microscope was set up as described by Pope et al*.* (2013), with TPF and DIC imaging capabilities (further details can be found in the supplementary Materials and Methods). 3D CARS images were taken as *z*-stacks over 50 μm depth in 0.5 μm steps, unless stated otherwise. CARS images in *xy* were taken with 0.1 μm pixel size, typically in a 100 μm×100 μm frame, with 0.01 ms pixel dwell time, and time-average total power of ∼20 mW at the sample.

Hyperspectral imaging is performed by changing the relative delay time between the equally linearly chirped pump and Stokes pulses, which results in a tuning of the instantaneous frequency difference across the entire 1200-3800 cm^−1^ vibrational range, owing to the large bandwidth of the Stokes pulse (Pope et al., 2013). In practice, scans were taken between 2600-3800 cm^−1^ in 5 cm^−1^ steps over a 10 µm×80-100 µm area, with 0.1 μm pixel size and a pixel dwell time of 0.001 ms, ten times less than the single frequency CARS images, to reduce photo-damage and motion artefacts. Raman-like spectra were retrieved from CARS spectra using hyperspectral image analysis (HIA) software developed in-house (Masia et al., 2015). Hyperspectral images were background-corrected by subtracting an image measured under identical excitation and/or detection conditions but with pump and Stokes pulses out of time overlap, then were noise-filtered using a singular value decomposition (SVD) algorithm on the square root of the CARS intensity to retain only components above noise. CARS intensity ratios were calculated by dividing the background-corrected CARS intensity by the corresponding non-resonant CARS intensity measured in glass under the same excitation and detection conditions. The phase-corrected Kramers–Kronig method (PCKK) was used to retrieve from the CARS intensity ratio the complex CARS third-order susceptibility (normalised to the non-resonant value in glass), which is linear in the concentration of chemical components (Masia et al., 2013). Shown spectra are the retrieved imaginary part of the susceptibility [Im(χ)] normalised to the total area, and Origin software (OriginLab) was used to plot these against eggs or embryos of the same developmental stage and/or conditions.

TPF imaging of lipid stains absorbing at ∼480 nm and emitting at 510 nm±40 nm, simultaneously with CARS, was possible using a third near-infrared beam centred around 930 nm, hence separated from the pump and Stokes beam, independently optimised in time domain to provide Fourier-limited pulses of ∼30 fs duration at the sample for maximum TPF excitation (see Pope et al., 2013). TPF was detected simultaneously with CARS in the wavelength range 498-540 nm, via appropriate dichroic beamsplitters and band pass filters (Semrock) in front of a second photomultiplier (Hamamatsu, H10770A-40). Autofluorescence images were taken simultaneously with TPF and CARS, and were detected in the wavelength range 451-487 nm using an appropriate dichroic beam splitters and band pass filters (Semrock) and a third photomultiplier (Hamamatsu, H10721-210).

Three-dimensional DIC images were taken with 20 ms frame exposure time and as *z*-stacks over the full cell-depth (∼70 μm) in 0.5 μm steps, using Micro-Manager software (Vale Lab, UCSF). A 12.5° polarisation angle in the de-Senarmont DIC illuminator was used, yielding a 25° phase offset, as this is found to be sufficiently small to enable good image contrast and sufficiently high to enable quantitative analysis of small phase objects such as lipid membranes (McPhee et al., 2013).

The MultiCARS software developed in-house was used to collect and regularise images from the CARS microscope. ImageJ (NIH) was used to assemble image stacks into maximum intensity projections and depth colour-coded projections.

### Intra-cytoplasmic sperm injection (ICSI)

ICSI was performed as described by Yoshida and Perry (2007). Some zygotes died after ICSI and these were discounted from our data. Zygotes were imaged in M2 medium with DIC and subsequently a single *xy* image was taken of each, before culturing in KSOM (Millipore) at 37°C, 5% CO_2_ under oil. DIC images of the embryos were taken five days later, and a score of the number of blastocysts in the population of living embryos was made. Control embryos were kept in M2 and not imaged, before culturing in KSOM. DIC images were then taken after development. Data are from five separate trials.

### Aggregate analysis

CCDPlot software developed in-house was used to find the 3D coordinates amplitude maxima and sizes of LDs from the single-frequency CARS images. The background signal was subtracted, and the square root of the data was calculated to give amplitudes proportional to the number of CH_2_ bonds in the sample. Peaks were fitted with a Gaussian function in three dimensions, and their amplitude, *xyz* width and centre was used to describe the LD. Origin was used to plot these parameters and analyse them statistically. An Origin script was used to assign an index to LDs separated from their nearest neighbour by less than a user-defined value *L*, the index being identical for all LDs belonging to the same cluster. The occurrence of this index was then used to calculate the number of LDs in each aggregate (called aggregate size) and the number of aggregates. We found that the optimum parameters to account for the observed clustering in the CARS images were obtained by calculating the distance between two LDs in resolution units, i.e.:

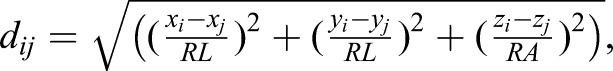
with *x*_i_,*y*_i_,*z*_i_ coordinates of the i^th^ LD, RL=0.3 μm and RA=0.6 μm being the lateral and axial CARS resolution, respectively, and using 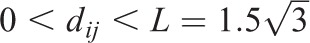, i.e. *L* equal to 1.5 times the distance in resolution units.

In order to compare mean aggregate sizes across different developmental stages, the probability P_k_ of each size k=1, 2, 3… (including size 1, i.e. isolated LDs) was calculated from the histogram of the aggregate size, as P_k_=O_k_/O, where O=Σ_k_ O_k_ and O_k_ is the occurrence of size k. The mean square size was then calculated as 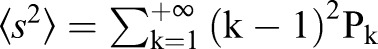 for each egg or embryo (where we subtracted 1 to obtain the number of partner LDs in an aggregate). Origin was used to plot the square root of 〈*s*^2^〉 
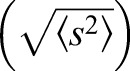
 for all eggs or embryos of a given developmental stage, against the total number of LDs at that stage.

HIA, MultiCARS and CCD plot (as executable) software can be obtained by contacting the authors.

### Statistical analysis

Correlation studies were carried out using an ImageJ ‘Correlation Threshold’ plug-in. The Pearson's correlation coefficient is calculated by coordinate correlation of pixels above a certain intensity threshold. Individual thresholds are calculated with ImageJ itself as intensities that give a Pearson's coefficient of 0. The resulting Pearson's coefficient for all pixels above threshold (RColoc) is a number between 0 and 1, to show the degree of significant correlation between CARS and TPF images. A correlation is accepted with a Pearson's coefficient of >0.5, but a significant correlation is considered with a coefficient of >0.95 (95% statistical confidence limit), as any lower value suggests the co-localisation seen is not more likely than random chance. The output correlation scatterplots show the linear relationship between pixels in corresponding CARS (*x*-axis) and TPF (*y*-axis) images.

### LD size analysis

In order to compare mean LD sizes across different developmental stages, the volume of each LD was calculated using V_i_= W_i*x*_W_i*y*_W_i*z*_, where W_i_ is the diameter of the i^th^ LD in each *xyz* dimension obtained from the 3D fitting procedure of the CARS amplitude using CCDPlot software. To account for subresolution droplets, the volume of each LD was normalised as V_in_=V_i_(A_i_/A_b_), where A_i_ is the CARS amplitude (square root of the CARS intensity) of the droplet, and Ab represents the amplitude of the brightest droplet in the egg or embryo. A LD effective diameter was then calculated as d_i_=(V_in_)^1/3^. The probability P_di_ of each diameter d_i_ was calculated from a histogram (see Fig. S1) of the diameters of LDs (bins of 1×10^–3^ μm) present within that particular egg or embryo, as P_di_=O_di_/N, where N is the total number of LDs and O_di_ is the occurrence of diameter d_i_. The mean diameter was then calculated as ⟨d⟩=Σ_i_ d_i_ P_di_ for each egg or embryo. Origin was used to plot the mean ⟨d⟩ of all eggs or embryos of a given developmental stage, against the mean total number of LDs at that stage.
